# Research Ready Grant Program (RRGP) protocol: a model for collaborative multidisciplinary practice–research partnerships

**DOI:** 10.1186/s12961-022-00870-x

**Published:** 2022-06-13

**Authors:** Tracy Flenady, Trudy Dwyer, Julie Kahl, Agnieszka Sobolewska, Kerry Reid-Searl, Tania Signal

**Affiliations:** 1 School of Nursing and Midwifery, Central Queensland University, Building 18, Bruce Highway, Rockhampton, 4702 Australia; 2 Central Queensland Hospital and Health Services, Canning Street, Rockhampton, 4701 Australia; 3 School of Health, Medical and Applied Sciences, Central Queensland University, Building 6, Bruce Highway, Rockhampton, 4701 Australia

**Keywords:** Research capacity-building, Programme evaluation, Research training

## Abstract

**Background:**

Little attention has been given to the process of implementing or evaluating a structured academic–clinician (university–health service) research capacity-building (RCB) model within healthcare settings. We have developed a model for collaborative multidisciplinary practice–research partnerships called the Research Ready Grant Program (RRGP). The RRGP is informed by Cooke’s (BMC Fam Pract 6:44, 2005) RCB framework and principles. The aim of the study outlined in this protocol is to conduct a process and outcome evaluation of the programme. We will explore how the RRGP's structured mentor model contributes to RCB of clinician-led multidisciplinary research teams. We will identify key factors at the organization, team and individual levels that affect research capacity of health professionals working in one regional health service district. This protocol describes the RRGP design and outlines the methods we will employ to evaluate an RCB programme, the RRGP, delivered in a regional health service in Australia.

**Methods:**

The study will adopt an exploratory concurrent mixed-methods approach designed to evaluate the process of implementing an RCB model across one regional hospital and health service. Both quantitative and qualitative data collection methods over a 12-month period will be implemented. Data triangulation will be applied to capture the complex issues associated with implementing collaborative multidisciplinary practice–research partnerships.

**Discussion:**

The RRGP is an innovative RCB model for clinicians in their workplace. It is expected that the programme will facilitate a culture of collaborative multidisciplinary research and strengthen hospital–university partnerships.

## Background

Healthcare clinicians’ engagement in practice-based research is necessary for improving the quality of healthcare and ultimately patient outcomes [2–4]. Practice-based research can be influential in informing and shaping healthcare policy and evidence-based practice [5, 6]. And yet, “the research–practice gap” remains a problem in healthcare [7, 8]. Clinician-led research is limited, and knowledge produced from research is not routinely translated into practice [9, 10]. This gap has negative implications for healthcare delivery. In Australia, reportedly, patients receive care that is deemed appropriate (based on best evidence) only 57% of the time [10]. The gaps in provision of evidence-based care is an equally significant problem in other Western countries [7, 10].

Developing the capacity of clinicians to engage in health research is a recognized strategy to respond to the research practice gap. According to the Australian Government [11] Review of Health and Medical Research, involving clinicians in research drives a continuous improvement mindset as the research is focused on identifying solutions to clinical problems. Clinician-led research also instils a sense of ownership of the research and a commitment to translate new evidence into practice [12]. There has been criticism of the traditional top-down approach to translating findings from non-clinicians’ research projects into clinical practice [6, 13]. The end users or the clinicians need to be included in the knowledge discovery phase to ensure that the research is applicable to practice and the context [13]. However, there is no established evidence base on how to best engage clinicians in conducting and leading research with literature in this evolving field [3].

The need for clinician-led research rhetoric is not, however, well integrated into workplace practice [2, 12, 14]. Cooke et al. [15] observe that the clinical areas most often cited as being of greatest need for increased research capacity are those with the lowest research skill and activity base. Cited barriers to conducting research include inadequate training in research methods [12, 16], lack of collaborators and support staff [17] and lack of organizational support and resources [16]. In the largest study of its kind in Australia, Hiscock et al. [18] surveyed 1027 clinicians. The participants identified protected research time (50%), designated research space (42%), clinical trial coordinators (35%), institutional funding (34%) and mentoring (33%) as critical enablers of research [18]. Hiscock et al. [18] conclude that to realize recommendations in the Australian Government review, hospitals need to actively facilitate conditions for clinician-led research.

The role of the healthcare organization as an enabling structure in fostering research cultures and environments is well recognized [2, 12, 19]. The organization’s enabling function includes provision of funding, sustainable resources and support including training [3, 19]. In their systematic review, Harding et al. [20] found that among healthcare organizations in the United States, United Kingdom and Germany, higher levels of research activity were positively associated with increased organizational efficiency, improved staff satisfaction, reduced staff turnover, improved patient satisfaction and decreased patient mortality rates. In other words, enabling research cultures can be thought of as a long-term investment that brings long-term gains for healthcare organizations.

Research capacity-building (RCB) is critical to promoting evidence-based healthcare delivery and continuous quality improvement [2, 3]. Holden et al. describe RCB as a “process of developing sustainable abilities and skills enabling individuals and organisations to perform high quality research” [14]. Researchers identify that RCB requires multifaceted and integrated approaches, including experiential learning and research translation activities [13, 21], as opposed to single interventions such as one-off training [3, 22]. Integrated approaches are reliant on leadership, organizational needs and management support which imply implementation of funded interventions [19, 21]. However, there is limited evidence reporting RCB models that successfully engage health professionals in research [3, 19].

Cooke’s [1] framework to plan and evaluate RCB in healthcare has been used in different practice settings internationally [3,23,24]. This framework can be applied at the individual (by participation), team (multi- and interprofessional involvement) and organizational (infrastructure and support) level [1]. Cooke’s [1] framework is based on six principles: developing skills and confidence; ensuring the research is close to practice; developing linkages and partnerships; developing appropriate dissemination; building elements of sustainability and continuity; and investment in infrastructure. Cooke [1] developed her framework through the blending of knowledge from analysis of the literature, policy documents and the experience of one Research and Development Support Unit in the United Kingdom [5]. Cooke et al. [15] used this framework to evaluate the “designated research team” approach to building research capacity in primary care in the United Kingdom. In their study, multidisciplinary research teams received a small grant to conduct research in the primary healthcare setting over a 2-year period and had access to various modes of training [23]. Cooke et al. [23] concluded that the framework can be useful as a basis to evaluate and compare various RCB projects.

While the Cooke et al. [15] study involved multidisciplinary teams of novice researchers, the focus was on evaluating the research outcome, rather than the process of multidisciplinary collaborations. Multidisciplinary collaboration is thought to generate team work, and it is an essential component of best practice in healthcare to maximize patient outcomes [25]. A multidisciplinary research team approach is also considered to generate a high degree of collaboration that can lead to further insights about the issue under study [26]. And yet, the literature on multidisciplinary research teams focuses on defining the term, rather than examining how they can be best activated to advance scientific knowledge and healthcare practices [27, 28]. There is limited research on the influence of multidisciplinary teams on research outcomes [26]. Importantly, Aboleala et al. [28] observe that the expectations and values of the team members regarding the multidisciplinary research process can vary, affecting research outcomes. Identifying the competencies and resources necessary for successful multidisciplinary contributions to science is an important foundation which could be used to guide a research design [28].

We will use the Cooke [1] framework to evaluate an RCB programme called the Research Ready Grant Program (RRGP), delivered in a regional health service in Australia. The programme will be delivered to clinicians who will form into self-selected multidisciplinary research groups at one hospital. We hypothesize that the RRGP is an RCB model that facilitates a culture of collaborative multidisciplinary research across all levels of the health service. This protocol describes the RRGP design and evaluation.

## Methods

### Research objectives

- To evaluate and report on the process of implementing a collaborative multidisciplinary RCB model in one regional health service district.
- To identify how the RRGP structured mentor role contributes to RCB of clinician-led multidisciplinary teams.
- To identify key factors at the organization, team and individual levels that facilitate successful implementation of the RCB intervention in one regional health service district.

### Setting

The study site is one Hospital Health Service (HHS) located in regional Queensland, Australia. This HHS provides public health services in hospitals and communities across an area of 117,000 km^2^ and in 2017, published their long-term healthcare strategy for the region. The strategy, titled Destination 2030: Delivering Great Care for Central Queenslanders, articulates a need for sustainable research partnerships between the hospital and local universities to promote translational research and mutual training focused on innovative healthcare practice [29]. The strategy recognizes the increased demand for health services in the region and the corresponding need to have the right health service infrastructure in order to provide evidence-based healthcare to effectively respond to the populations’ health needs. Promoting sustainable research partnerships is recognized to enhance community health outcomes. The RRGP is a 3-year initiative that emerged from the strategy and is embedded within the strategy’s vision and objectives. The RRGP, led by Central Queensland University, has been developed as a partnership between the HHS, Central Queensland University and the University of Queensland’s Rural Clinical School.

### Participants and participant recruitment

There are five convenience groups:

#### Group 1

Group 1 comprises people directly involved in the development, delivery and implementation of the RRGP. Group 1 includes the RRGP project manager, project officer and members of the RRGP working party. The RRGP working party comprises senior researchers from participating universities and the hospital and senior decision-makers from the hospital.

#### Group 2

Group 2 consists of research facilitators—academics from participating universities and health service staff with research expertise. Research facilitators are responsible for facilitating weekly workshops to the research teams of clinicians (maximum number of four teams per research facilitator) over a period of 8 weeks. Eligible research facilitators have research expertise and experience that align with the proposed research topics of the teams assigned to them. Research facilitators are invited to participate by representatives from the RRGP working party and must meet predetermined criteria (PhD qualification) to be eligible. As the research facilitator positions are funded, there is capacity for a maximum of six research facilitators to be recruited each year the programme runs.

#### Group 3

Group 3 comprises the research mentors—academics from participating universities and HHS staff who are suitably qualified (PhD, research outputs and grant income). Research mentors are specifically recruited by the RRGP working party to align with the research topics of the RRGP teams who successfully matriculate from phase 1 of the RRGP to phase 2; phase 1 being the 8-week education workshops, and phase 2 being the operationalization of the successfully funded projects. Each research mentor is responsible for providing one team with ongoing support for the duration of their project, usually limited to 10–12 months. A memorandum of understanding is drawn up between the research mentor and the RRGP working group that sets out the level of support that is agreed upon and expected from the mentor. The research mentor position is funded, with mentors only able to claim payments once specific (agreed upon) milestones are met. Milestones include proof of ethics submission and submission of an interim, midway and final project report. Eight projects are allocated for in the annual budget, so therefore, up to eight research mentors are recruited annually. The RRGP has developed specific criteria that researchers (both academic and industry) are to meet before they are eligible to fulfil the research mentor role.

#### Group 4

Group 4 consists of weekly guest lecturers, topic experts from the university or hospital, who are tasked with developing and delivering the weekly lectures throughout phase 1 of the programme. See Table 1 for details around topics covered. This cohort is required to be suitably qualified according to existing RRGP criteria (tertiary qualified educators) and are recruited by the RRGP working group to fulfil these funded positions.

**Table 1:** RRGP 8-week workshop schedule

TOPIC:Formulating a research problem			How this topic informs the project proposal	Applied activities worksheet
Week 1 24 February	Introduction to the RRGP 30 min	Welcome on behalf of Central Queensland Hospital and Health Service (CQHHS), Central Queensland University (CQU) and University of Queensland (UQ) Destination 2030 Overview of the RRGP		
	Refining the problem Formulating a research question; 30 min	List explicit goals and objectives for your research project and state the hypothesis/research question List the logical arguments that you would like to use to convince the reviewers that your proposed research project is important and relevant (template will be provided at the workshop to help facilitate this)	Identifies the research problem Identifies the research question	Confirm research question via communication with your team
TOPIC: Formulating a research question searching and reviewing the literature			How this topic informs the project proposal	Applied activities worksheet
Week 2 3 March	Searching and reviewing the literature	Develop a search question using PICO [Patient/Problem, Intervention, Comparison, Outcome] and CKN [Clinical Knowledge Network] frameworks. Learn how to produce an outline for the literature review that outlines a rationale/justification of how your team’s project will fill any gaps, contribute to the field of research or contribute to existing or improved practice	Background (literature review)	PICO template provided for teams to develop a search strategy specific to their project
TOPIC: Conceptualizing the research design			How this topic informs the project proposal	Applied activities worksheet
Week 3 10 March	Research Design Various methods	Develop an understanding of research designs (quantitative, qualitative, mixed methods) so that your team can match your research question with the appropriate research design	Project design Methodological approach	Research design canvas template provided for teams to explore their research question in terms of identifying the most appropriate design. Research proposal template provided
TOPIC: Population/participants			How this topic informs the project proposal	Applied activities worksheet
Week 4 17 March	Research design Participants/population	Develop an understanding of population/participants: Who are they? Where will I find them? Recruitment and consent process, inclusion/exclusion criteria and potential for risk, burden and benefit to the participants	Project design Participants/population	Worksheet to aid the identification of the project’s population/participants
TOPIC: Data collection			How this topic informs the project proposal	Applied activities worksheet
Week 5 24 March	Research design Quantitative/qualitative: sampling, sample size, power	Develop an understanding of sampling, sample size, data collection methods and tools. How will your team collect the data?	Project design Data collection techniques	Worksheet to aid the identification of the project’s sampling collection. Include as much information about sampling as you can
TOPIC: Data analysis			How this topic informs the project proposal	Applied activities worksheet
Week 6 31 March	Research design Quantitative/qualitative: data analysis	Develop an understanding of data analysis. How will your team measure, manipulate and/or analyse the information that you collect?	Project design Data analysis techniques	Quantitative/qualitative worksheets to aid the planning of your data analysis. Be as detailed as possible
TOPIC: Presenting your topic			How this topic informs the project proposal	Applied activities worksheet
Week 7 7 April	Elevator pitch – present your project to the group	Develop an understanding of how to present your project	Dissemination strategy Project presentation skills	Presentation slide templates
TOPIC: Ethical considerations			How this topic informs the project proposal	Applied activities worksheet
Week 8 14 April	Ethics and research	Identify where and how to access human research ethics committee (HREC) documents and other ethical issues related to your proposed study (risk, benefit, etc.) Understand the essential elements completing the HREC and site-specific assessment (SSA) and how to assess the quality of a study	Ethical considerations, including code of conduct and risk management Research integrity and practices to maintain integrity	Research proposal template populated. Access and download the appropriate ethics application and complete your application

#### Group 5

Group 5 comprises clinicians who are interested in doing research and have enrolled in the RRGP. Participation in the RRGP is open to all staff employed at the regional HHS. Staff are recruited via internal emails and social media snowballing. Potential participants are required to complete an application form in which they outline a specific clinical issue, quality improvement idea or a patient safety issue that they are interested in researching. In addition, participants are required to self-select into multidisciplinary research teams prior to commencement of the programme. If potential participants express interest to participate but do not have a specific research topic, they are encouraged to join other research teams. The submitted applications are assessed against selection criteria presented on the application form. Clinicians whose applications are successful will be invited to participate. Successful applicants are also required to commit to having at least one team member present for each of the weekly workshops. The workshop presentations are offered outside business hours once a week over an 8-week period.

### RRGP design

The RRGP combines a structured education programme with a research mentorship model that supports the development, implementation and evaluation of small research projects. The RRGP is a peer-reviewed, merit-based programme with aims that align with Cooke’s [1] RCB principles. The programme aims to (1) build the research capacity and skills of clinicians at one Queensland HHS; (2) strengthen partnerships between tertiary learning organizations and health services; (3) promote evidence-based practice; (4) facilitate development of quality research; (5) disseminate research findings; and (6) encourage novice researchers and clinicians in developing a research career. The programme comprises two phases: skills development lectures and workshops (8 weeks) followed by successfully funded teams’ operationalization of their research project (10–12 months).

#### Phase 1—skills development workshop

Phase 1 comprises eight skills development workshops designed to increase research knowledge. The workshops are delivered weekly over 8 weeks with the participants engaging face to face in 1-hour-long lectures followed by 2-hour workshop sessions. The lectures are delivered by experienced presenters who are chosen by the RRGP working group for their topic expertise. Following each lecture, the participants, along with their respective research teams, attend a 2-hour workshop facilitated by research facilitators. The research facilitators assist the research teams to apply information presented in the weekly lecture to their specific research topic/idea. The research facilitators help to refine research questions and methodologies, guiding the participants in the development of a final research proposal.

The skills development workshops cover key topics related to the research process and formulating a research proposal. Table 1 presents an outline of the weekly topics and articulates the learning outcomes. The content for the skills development workshops captures the steps involved in the ethics submission process resulting in detailed project proposals.

At the conclusion of the 8 weeks, research teams are expected to have been presented with sufficient information and support to develop a grant application for their specific project. While optional, the teams are encouraged to submit their research proposal to the RRGP working group for merit-based funding up to the value of 7000 Australian dollars per group. The applications undergo a blinded, peer review process and are assessed according to predetermined criteria that are shared with the participants. Funding is awarded to the top eight applications, and these groups are then assigned a dedicated research mentor for the duration of the research project phase.

#### Phase 2—the research project

Research teams whose proposals get approved proceed to phase 2 where they conduct their research over a period between 10 and 12 months. As research supervision and mentorship are intrinsic to the successful completion of research projects [30], the research teams work with a dedicated research mentor. The role of the research mentor in the second phase of the RRGP is to support the implementation, evaluation and reporting of the final research project.

The successfully funded teams, as grant recipients, are required to meet ongoing project milestones including ethics submission, project progress reporting and dissemination goals. Teams are also required to deliver a final report to the RRGP working group. The grant recipients are also expected to present their research at the annual HHS Research Showcase Day and are encouraged to disseminate findings through publications, conference presentations and/or to influence policy change. The research mentor, as a team member, is offered a pre-negotiated authorship position on any publications arising from the research. Authorship order on papers will be negotiated at the outset of the project and will reflect the relative intellectual contribution to the project by all parties as outlined by National Code of Conduct for Research.

### RRGP evaluation

The Cooke [1] framework for RCB will be employed to guide the evaluation process. The framework has been shown to be useful as an evaluation framework of RCB initiatives [15]. Table 2 shows the application of Cooke’s framework to the RRGP. As can be seen, the RRGP interventions and the measurements through which they will be evaluated are directly linked to the six RCB principles proposed by Cooke.

**Table 2:** Application of Cooke’s RCB framework to the RRGP

RRGP RCB principles	RRGP intervention	RRGP measurements
Research capacity is built by developing appropriate skills and confidence through training and developing the opportunity to apply skills in practice	Delivery of 8-week skills development workshop	Number of initial applications and developed proposals Attendance at the eight weekly lectures Participant evaluation of their experience of the programme (survey) Interviews with participants
RCB should support research “close to practice” to highlight its usefulness to practice	An industry-driven research problem/topic is a requirement for admission to the RRGP Ongoing support from research facilitators throughout phase 1	All expression of interest (EOI) submissions for admission to RRGP address clinician-identified problems and are consistent with the CQHHS Destination 2030 vision
Developing linkages, partnerships and collaborations enhances RCB	Research skills development workshops Connecting with experienced researchers/ research mentors Multidisciplinary team participation is a requisite for admission to the RRGP phase 1 and phase 2 Weekly unstructured networking opportunity	All EOI submissions for admission to RRGP list at least two disciplines Reporting on the number of emergent collaborative partnerships (individual, team and organizational levels) Interviews with the mentors/facilitators/participants Multidisciplinary participation evident on phase 2 grant submissions
RCB should ensure appropriate dissemination	Elevator research pitch Central Queensland Hospital and Health Service (CQHHS) Research Showcase External dissemination: conference presentations, journal article	Annually, the number of: Teams who present an elevator research pitch to colleagues Funded teams who present an oral or poster presentation at the annual HHS research showcase Publications accepted in peer reviewed journals External conference presentations delivered RRGP project report developed and disseminated
RCB should ensure elements of continuity and sustainability	Grant round (RRGP phase 2) Development of programme resources Online resource development	Annually, the number of: RRGP grant submissions RRGP grants awarded—funding-dependent RRGP projects completed—ongoing Programme resources and templates developed Online resources developed
Developing appropriate infrastructure enhances RCB	Establish sustainable an ongoing model for delivering the RRGP Extend reach to settings other than Rockhampton	Successful funding for future years Ongoing commitment to the RRGP model Identify platform to store developed RRGP resources Implement strategies for rural/remote clinicians to participate in phase 1

The RRGP will be evaluated at different stages of the project cycle. First, the initial RRGP applications will be examined to gain a sense of how the programme can develop skills and confidence of clinicians to conduct research. At the completion of the skills development workshop, the developed research proposals will be assessed in relation to whether the RRGP training and the respective opportunities to apply research skills in practice contributed to development of the basic research skills. A document review will be conducted of applications developed by the participants.

The participant responsiveness to the RRGP will also be measured. An audit of the de-identified attendance sheets will be undertaken to calculate the number of participants who completed the skills development workshop. In addition, programme records of the composition of research teams, their disciplines and research topics will be examined.

Individual semi-structured interviews will be conducted with RRGP participants and abovementioned groups 2, 3 and 4 after each annual cycle of the RRGP. The development of the interview schedules will again be guided by Cooke’s RCB framework. The interviews will explore participants’ perceptions and experiences of the workshops with a focus on how the workshops enable the novice researchers to develop skills and confidence, as well as linkages, partnerships and collaborations. Open comments will also be encouraged related to the programme and participants’ expectations.

Participants will also complete a survey at the end of the programme which has RCB measures. The survey adopted in this study is the validated research capacity and culture (RCC) tool developed by Holden et al. [14]. This survey is specifically designed to measure research capacity and culture across three domains: organization, team and individual. The RRC tool has been successfully tested in Queensland health facilities and has a reported good internal consistency for organization, team and individual domains (alpha = 0.95, 0.96 and 0.96, respectively). It consists of a series of statements where participants rate their response on a Likert-style scale of 1–10 with 1 being the lowest skill or success level and 10 being high success/skill. The final survey used in our study consists of demographic data, 51 RCC domain questions (organization *n* = 18; team *n* = 19 and individual *n* = 14) and an open-ended response section, designed to elicit specific contextual information.

During the first round of the programme, we will evaluate the structure and content of the programme to inform the quality of its subsequent delivery. Vijn et al. [31] assert that the design-based research can be risky due to uncertainties in participant behaviour and circumstances in the learning environment. The plan-do-study-act method (PDSA) [32] will be applied to evaluate and optimize the workshop. PDSA, as a quality improvement strategy, enables fast implementation and quality improvement of healthcare interventions in healthcare [31]. During a PDSA cycle, the programme will be planned, performed, evaluated and improved. The process will be evaluated through three rounds of focus groups with the expert researchers who fulfilled the role of research facilitator, to be conducted in the beginning (after the workshop has started), middle and the end of one round of the RRGP. The focus will be on assessing the aspects of the workshop that are working well and those that require improvement. The topic guides of the focus groups will revolve around the experience of the research facilitators guiding groups of novice researchers to prepare research proposals. The reiterative nature of the focus groups will also enhance respondent validation [33]. The results of the evaluation will be used to improve the design of the skills development workshop.

At the latter stage of the programme, the impact of the RRPG will be examined. Dissemination of the research findings, continuity and sustainability of the research projects will be assessed through the number of grants awarded, as well as conference presentations and journal papers. A review will also be conducted of the potential media reports documenting the research projects.

### Data analysis

This study will adopt an exploratory concurrent mixed-methods approach designed to evaluate the implementation of the RRGP. The study comprises both quantitative and qualitative data collection methods for each 12-month period that the programme runs. Data triangulation has been adopted to capture the complex issues associated with implementing collaborative multidisciplinary practice–research partnerships. Data triangulation will also enhance the confirmability and credibility of the findings [34].

#### Survey

Descriptive data will be used to report the participants’ demographic responses which include age, professional stream and employment status. Statistical analyses will be performed using SPSS. The RCC domains (organizational, team or individual) will be summarized using descriptive statistics [median, interquartile range (IQR)] and median scores categorized as low, medium or high. The Friedman test [35] will determine the difference in success/skill between the three domains, and post hoc analyses will be conducted to determine where differences have occurred. An exploratory factor analysis will be conducted to determine underlying themes of the three domains. Correlation analyses will be performed to identify any relationship between demographic data and the identified factors. To determine the internal consistency of all domains and identified factors, Cronbach alpha analysis will be completed. The level of significance will be set at *P* < 0.05.

#### Open-ended responses and interviews

Open-ended responses will be transcribed, word for word and entered onto an Excel spreadsheet for analysis. Research team members will independently read and analyse the responses using content analysis [36, 37]. Researchers will meet to reach consensus of first-level analysis. This approach was selected as it is a practical approach that permits the presentation of results in everyday language, facilitating accurate interpretation and adoption by wider audiences [38]. Finally, findings will be presented to other team members, not involved with the initial stages of analysis but who are familiar with the topic, who will evaluate the findings to ensure they match reality.

Thematic analysis will be conducted on the de-identified transcripts from interviews and focus groups [36]. Again, the researchers will independently read and analyse the transcripts. They will then agree on a coding framework that will be developed. Qualitative data analysis will be performed with the assistance of NVivo.

#### Document review

An audit will be conducted of a range of documents that will be developed in the course of the RRGP.

## Discussion

This protocol presents a framework for implementing and evaluating the RRGP which aims to build capacity of clinicians to conduct research close to practice. The programme is designed to offer support and skill development for clinicians to conduct quality research. The programme is developed in alignment with the HHS’s strategy focused on promoting translational research to enhance innovative healthcare. Our evaluation will identify key factors at the organization, team and individual levels that affect research capacity of health professionals. We will also apply Cooke’s [1] framework to explore how the individual, team and organizational levels interact together in the context of the RCB initiative in one healthcare organization. This project will contribute to the empirical knowledge about RCB initiatives for clinicians to facilitate clinician-led research. It will provide information about enablers and barriers to conducting research that is close to practice within multidisciplinary research teams. Our findings have the potential to guide future initiatives to engage health professionals in quality research.

The RRGP is developed and implemented based on the premise that engaging clinicians in research can lead to production of translational research [13]. The programme is designed to upskill clinicians to conduct research on practice-related issues and then be able to disseminate the findings. Designing and conducting research and later the dissemination process rely on collaboration between the clinicians and their academic facilitators and mentors. Academic and professional collaborations have the potential to increase research productivity and quality, improve learning and enhance the development of new skills across partnerships [39]. The involvement of a mentor is also valuable in the writing-for-publication stage that requires a specific style and standard and the use of technical skills that may seem unattainable to novice researchers [40]. We will examine the role of mentors and facilitators in building research capacity of clinicians. There is a paucity of literature unpacking the role and how it can be utilized to support clinicians as individuals and groups in doing research. The evaluation can potentially illuminate the mechanisms for engaging clinicians in production and dissemination of knowledge relevant to practice.

The significance of the RRGP is that it adapts a multidisciplinary research team approach. While multidisciplinary healthcare delivery is presented as the golden standard in healthcare delivery, its delivery is difficult due to professional silos and practice differences [41–44]. There is also limited research on how multidisciplinary research teams effectively work [26]. We anticipate that this study will provide some important insights on how multidisciplinary teams can enhance the research processes.

### Limitations

As the RRGP evaluation will only occur in one setting, the transferability of the results to other settings will be limited [31]. To account for this limitation, we intend to develop theoretical principles to contextualize the RRGP framework. Further, we will provide in-depth descriptions of all five participant groups, clear overviews of the evaluation and analysis methods used and the context of the learning environment of the programme to enable comparison of our results to other settings.

We recognize that time-limited initiatives such as the RRGP are limited in scope to fully implement the six principles of RCB proposed by Cooke [1]. Our outcome measures include the number of submitted grant applications, evidence of multidisciplinary projects, peer-reviewed journal submissions and conference presentations. However, besides these traditional outcome measures, Cooke [1] highlights the need to disseminate the social impact of research (impact on the lives of patients, for communities, and quality of services). Pearson et al. [45] argue that closing the research–practice gap involves multiple phases and closing three knowledge translation gaps. The first gap exists between the need for knowledge and the discovery of that new knowledge [13]. The second gap is situated between the discovery of new knowledge and the clinical application of that knowledge which requires that the clinicians translate the findings and integrate them into their practice [13]. The third gap is positioned between the clinical application and the development of routine clinical actions or policy. The RRGP can be said to target the first and the second gap. The clinicians are engaged in the discovery of new knowledge that is needed. The research findings can then be used in an endeavour to close the second gap. However, it is outside the scope of this evaluation to measure how successfully the programme can fully close the research–practice gaps.

According to Cooke et al. [15], RCB should ensure elements of continuity and sustainability. Research has shown that sustainability is an implementation issue which cannot be achieved through clinical projects alone [6]. Instead, ongoing commitment by the organizations to develop research cultures that generate research that is useful is required [19]. The RRGP is designed to develop these foundations. However, sustainability will require further interventions and funding focused on the health services having a strong ownership and investment in research development initiative. The graduates of the RRPG can have an active role in the future delivery of the RRGP and fulfil the role of mentors.

## Conclusion

Informed by Cooke’s [1] RCB framework and principles, we have developed a model for collaborative multidisciplinary practice–research partnerships—the RRGP. Our aim is to conduct a process and outcome evaluation of the programme to explore how the RRGP's structured mentor model contributes to RCB of clinician-led multidisciplinary research teams. We anticipate that our findings will contribute to the empirical knowledge about RCB initiatives for clinicians to facilitate clinician-led research. It will provide information about enablers and barriers to conducting research that is close to practice within multidisciplinary research teams. Our findings have the potential to produce new knowledge about formal mentoring programmes for multidisciplinary research teams and may be used to direct future clinical research engagement and capacity-building research activity and funding.

## Data Availability

As this is a protocol, there is no data at this stage.
